# Genome Sequence of the Soil Bacterium *Janthinobacterium* sp. KBS0711

**DOI:** 10.1128/genomeA.00689-15

**Published:** 2015-06-18

**Authors:** William R. Shoemaker, Mario E. Muscarella, Jay T. Lennon

**Affiliations:** Department of Biology, Indiana University, Bloomington, Indiana, USA

## Abstract

We present a draft genome of *Janthinobacterium* sp. KBS0711 that was isolated from agricultural soil. The genome provides insight into the ecological strategies of this bacterium in free-living and host-associated environments.

## GENOME ANNOUNCEMENT

Soil microbial diversity is shaped in part by bacterial secondary metabolite production (1). A well-characterized example comes from *Janthinobacterium* (*Betaproteobacteria*, *Oxalobacteraceae*), a purple-pigmented chemoorganoheterotroph that is commonly found in soils (2). The characteristic pigmentation of *Janthinobacterium* is due to the production of violacein, an antimicrobial compound that affects predator-prey interactions (3) and microbial-host symbioses (4). To date, only a few *Janthinobacterium* spp. have been sequenced. Despite a recent genome from lake sediments (5), most sequenced isolates come from either extremely cold or synthetic habitats (6–8). Here, we present the draft genome of *Janthinobacterium* sp. KBS0711, which was enriched from never-plowed soil at the Kellogg Biological Station Long-Term Ecological Research site (9, 10).

DNA purified from a single colony was prepared with the Illumina TruSeq DNA sample prep kit using an insert size of 250 bp and sequenced on an Illumina HiSeq 2500 using 100-bp pair-end reads (Illumina, San Diego, CA). Raw FASTQ reads were processed by removing Illumina TruSeq adaptors and the first 10 bp using Cutadapt (version 1.7.1) (11), interleaving reads using Khmer (version 1.3) (12), and quality-filtering with an average Phred score of 30 using the FASTX-toolkit (version 0.0.13, Hannon Lab [http://hannonlab.cshl.edu/fastx_toolkit/]). Coverage was normalized to 25 based on a k-mer size of 25 bp using Khmer. We removed NCBI-flagged coding regions using BWA (version 0.7.10) (13).

A total of 3,130,492 unmapped reads remained after quality filtering. We assembled the genome using Velvet (version 1.2.03) (14) with the following parameters: a k-mer size of 55, expected coverage of 18, and a coverage cutoff of 2.29. Contigs larger than 200 bp were annotated using Prokka (version 1.10) (15), and we predicted metabolic and physiological functions using MAPLE with bidirectional best-hit matches (http://www.genome.jp/tools/maple/) (16).

The draft assembly of *Janthinobacterium* sp. KBS0711 is 6.08 Mbp. It consists of 25 contigs with an *N*_50_ of 882 kbp, and a G+C content of 62.7%. Annotation detected 5,386 coding sequences, 1 rRNA, 69 tRNAs, and 1 transfer-messenger RNA (tmRNA). The 16S rRNA has 99% sequence identity to a *Janthinobacterium lividum* isolate (NCBI accession no. NR_026365.1).

In addition to the pathway for violacein production (VioA-VioD), we detected genes encoding traits that are associated with violacein production, including quorum sensing (QseC-QseB) and biofilm production (BdcA, KinB-AlgB) (17–19). The genome also contains genes that may allow the isolate to contend with nutrient starvation (PhoR-PhoB) and osmotic stress (EnvZ-OmpR). Last, *Janthinobacterium* sp. KBS0711 contains genes related to nitrogen (urea transport, dissimilatory nitrate reduction), phosphorus (phosphonate transport), and sulfur (sulfonate transport, assimilatory sulfate reduction) metabolism, which are important for soil functioning.

### Nucleotide sequence accession numbers. 

This whole-genome shotgun project has been deposited in DDBJ/EMBL/GenBank under the accession no. LBCO00000000. The version described in this paper is the first version, LBCO00000000.1. The code used for assembly is available online (https://github.com/LennonLab/JanthinoKBS0711).
